# Ethnopedology in the Study of Toponyms Connected to the Indigenous Knowledge on Soil Resource

**DOI:** 10.1371/journal.pone.0120240

**Published:** 2015-03-19

**Authors:** Gian Franco Capra, Antonio Ganga, Andrea Buondonno, Eleonora Grilli, Carla Gaviano, Sergio Vacca

**Affiliations:** 1 Dipartimento di Architettura, Design e Urbanistica, Università degli Studi di Sassari, Via Colombo n° 1, Nuoro, Italy; 2 Dipartimento di Architettura e Disegno Industriale “Luigi Vanvitelli”, Seconda Università degli Studi di Napoli, Abazia San Lorenzo ad Septimum, Borgo San Lorenzo, Aversa (Caserta), Italy; 3 Dipartimento di Scienze e Tecnologie Ambientali, Biologiche e Farmaceutiche, Seconda Università degli Studi di Napoli, Via Vivaldi n° 43, Caserta, Italy; 4 Dipartimento di Scienze Chimiche e Geologiche, Università degli Studi di Cagliari, Via Trentino n° 51, Cagliari, Italy; NERC Centre for Ecology & Hydrology, UNITED KINGDOM

## Abstract

In taking an integrated ethnopedological approach, this study aims to investigate the meaning of the distribution of the toponyms used in traditional and recent cartography of Sardinia (southern Italy). It is particularly, but not only, focused on those related to soil resources. Sardinia is particularly interesting in this respect, as its unique history, geography, and linguistic position makes it one of the Italian and Mediterranean regions with the greatest number of toponyms. This research investigated the toponyms belonging to an important sub-region of Sardinia, called Ogliastra (central-eastern Sardinia). The research was conducted through the following integrated approach: i) toponymy research and collection from different sources; ii) database creation and translation of toponyms from the Sardinian language (SL); iii) categorization of toponyms; and iv) graphical, statistical, and cartographic data processing. Distribution and diversity of toponyms were assessed using the compiled database, coupled with a geographical information system (GIS). Of around 7700 toponyms collected, 79% had already been reported in SL, while just 21% were in Italian. Of the toponyms in SL, 77% are of known meaning and 54% of these toponyms were characterized by a meaning directly and/or indirectly connected to specific environmental features. On the whole, morphology would appear to be the primary environmental factor able to explain the complex, articulated presence, distribution, and typology of the investigated toponyms. A least squares regression analysis of pedodiversity vs. topodiversity shows a very closed distribution, with an impressive high correlation index (R^2^ = 0.824). The principal factor analysis (PFA) shows that such a connection may be morphologically based, thereby confirming that pedodiversity and topodiversity are strongly linked each other. Overall, the research shows that an integrated ethnopedological approach, combining indigenous and scientific knowledge may be of great interest in order to mitigate the impressive phenomena of the indigenous knowledge lost.

## Introduction

Ethnoecology [1] is the cross-cultural study of how people perceive and manipulate their environments. It has traditionally focused on linguistic analyses of terms for plants, animals, habitats, and other ecological phenomena in a bid to reveal underlying structures of the human mind that influence human behaviour [2]. One of the first approaches taken in ethnoecology studies is to assess what natives consider as "worth attending to" [3] in their daily relationships with the environment.

As a part of ethnoecology, ethnopedology [4], is a hybrid discipline structured as the combination of natural and social sciences, such as soil science (including geopedological surveys), social anthropology, rural geography, agronomy, and agro-ecology [5]. Ethnopedology aims to document and increase understanding of local approaches to soil perception, classification, appraisal, use, and management [6]. This discipline deals with the consideration that pedodiversity is strongly related, not merely to physical-chemical behaviour and soil taxonomic properties, but also to anthropic management, historical uses, cultural practices and, indeed, the whole of the indigenous knowledge that characterizes a specific area.

From an international perspective, ethnopedological studies mainly regard Africa, America, and Asia, covering more than 60 countries and around 200 different ethnic groups [6]. By contrast, Pacific areas and Europe have been less investigated. Specifically, of all tropical and European areas, the Pacific and the Mediterranean are the most neglected, although they are characterized by an important rural population, many ethnic groups and amongst the greatest level of linguistic diversity. For example, as concerns the Pacific areas, out of a worldwide total of 5000 different endemic languages, 1600 are spoken in the Pacific islands [6]. In examining the Mediterranean, in the eleven European countries directly overlooking the Mediterranean sea, approximately twenty different primary ethno-cultural areas are recognized.

Toponymics or toponymy (from the Greek *tópos* meaning “place” and *ónoma* meaning “name”), a branch of onomasticsis (the study of names of all kinds), is the study of place names (toponyms), their origin, meaning, use, and typology [7]. As reported by Siderius and de Bakker [8], an ethnopedological approach can help better understand the connection of local field and place names with soil resources. Indeed, as argued by Elerie and Spek [9] toponyms are an excellent research topic at the interface between local/rural and expert/scientific knowledge, containing many elements gathered from the physical, social, and inner dimension of the landscape.

Of all Mediterranean regions, the island of Sardinia (southern Italy) is one of the most interesting areas, for several historical, geographical, and linguistic reasons. Quantitatively, the territory as a whole has around 200,000 place names [10], making for more than 8 toponyms/km^2^. In comparison with other Italian regions and Mediterranean areas characterized by neo-Latin languages, the Sardinian toponymy heritage is noticeably more conservative, since many toponyms continue to be reported, used, and known in their original or pseudo-original form, despite the changed linguistic, historic, and cultural conditions. Indeed, many Sardinian toponyms are of pre-Latin and pre-Punic origin [11]. For instance, in some sub-regions of Sardinia, the percentage of such indigenous pre-Romanic toponyms can be as high as 50%, as compared with a European mean of 2% of prehistoric toponyms [12].

From a linguistic point of view, such indigenous toponyms represent an example of the “*Sa Limba Sarda*”, *i*.*e*. “The Sardinian Language” (SL), a Romance [13] or archaic neo-Latin language [14]. Generally speaking, the term SL is used to group together all vernacular linguistic varieties spoken in Sardinia [15]. Among the tongues of Latin origin, SL is considered the most characteristic of all Latin languages since it represents the best preserved traits and words from the mother tongue, including lexical and phonetic factors, as well as morphological aspects [13]. At the same time it also reveals many other influences, including Phoenician, Catalan, Spanish, and Italian, bearing witness to the island’s rich history and the numerous rules that Sardinia has been subjected to [13–14]. Linguists recognise two main language varieties (*Logudorese* and *Campidanese*), yet SL is actually fragmented into numerous dialects and sub-varieties, which vary considerably from zone to zone and often even from one town to the next. Indeed, although the morphological and syntactical differences between *Logudorese* and *Campidanese* are not substantial, every major variety of Sardinian parlance has its own grammatical and orthographical system [16] in addition to significant phonological differences [17]. The fragmentation of the SL is the consequence of various, complex events that have characterized Sardinia’s history [14]. It is not only the result of the numerous invasions and waves of domination (Phoenicians, Romans, Vandals, Byzantines, Aragonese and Catalan Spanish, and finally mainland Italians), but also of the geographic and cultural isolation particularly applicable to the central areas of Sardinia [14], [18–21] such as Barbagia and Ogliastra.

From a sociolinguistic point of view, SL is a minority language mainly spoken in the villages and in private life. Most Sardinians are bilingual, speaking both SL and Italian [22], which today is the main language used in the cities and almost the only language used for official purposes. SL is also one of the fifteen European minority languages with more than one million speakers [23], but it suffers from a serious decline of language ability from one generation to the next [24]. This is why SL is considered an endangered language by UNESCO [25], a fact that urges core and applied studies on the multifaceted uses of SL, including through an ethnopedological approach. Indeed, linguistic diversity parallels ethnopedological richness [6], since oral tradition conveys local wisdom and knowledge on environmental resources from one generation to the next [26]. This means that as many endemic languages are threatened with disappearance, ethnopedological knowledge will also become lost [6].

This study aims to take an integrated ethnopedological approach to investigate the meaning of the distribution of the toponyms used in traditional and recent cartography of the region of Sardinia (southern Italy). It is particularly, but not only, focused on those related to soil resources.

## Materials and Methods

### Ethics statement

No written permission or authorisation was required to conduct this research. All the investigated areas are freely accessible and not restricted for research purposes. This research did not involve any endangered or protected plant species.

### Study area

**Fig 1:**
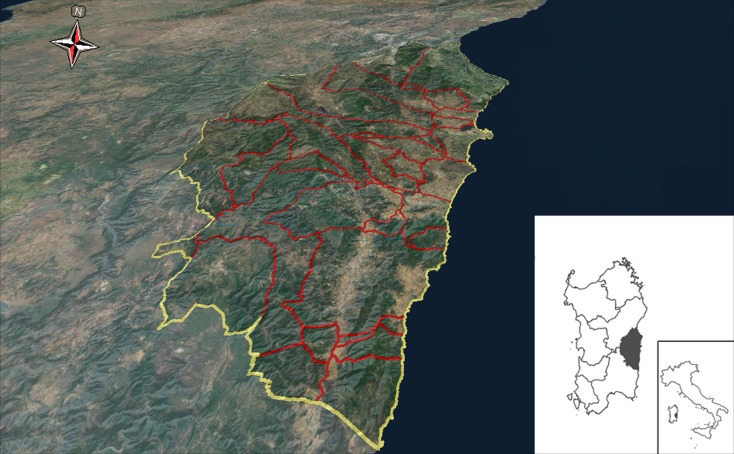
Location of the study area.

The island of Sardinia (southern Italy) lies in the Mediterranean Sea covering an area of 24,090 km^2^. This research investigated a sub-region of Sardinia (Fig. 1), called Ogliastra (9°16’- 9°44’ E, 39°32’- 40°13’ N). Indeed, together with Barbagia, Ogliastra (central-eastern Sardinia) is the sub-region that best preserves the original traits and words of the Sardinian language [13].

Ogliastra is mainly hill country, both inland and along the coast, with just a few plains characterizing the coast. It numbers 23 small municipalities of which just two consist of more than 4000 inhabitants. With around 58,000 inhabitants and an area of 1856.6 km^2^ [27], it is the Italian province with the lowest population density (31 inhabitants/km^2^). By way of comparison, the provinces of Cagliari (the capital of Sardinia) and Rome (the capital of Italy) have population densities respectively as high as 121 and 751 inhabitants/km^2^.

### The Sardinian language

In order to fully understand why SL has been better preserved in Ogliastra and why it represents a unique idiom, with no comparison with any other languages worldwide, S1 Table gives a brief overview of Sardinia’s history. Clearly, SL characteristics are strictly connected with the historical vicissitudes of Sardinia, which influenced its development. Indeed, even if the SL is, of all Latin origin languages, the one that best preserves traits and words from the mother tongue, it also reveals many other historical influences. Doubtless, the entire history of the SL development highlights the great political influence that conquerors have on both language and local knowledge.

The roots of the Sardinian language (sometimes referred to as Paleo-Sardinian, *i*.*e*. the SL spoken before the Roman dominion and the subsequent latinization), remain still obscure. Indeed, there are often substantial differences between theories about the development of SL, hence opposite results have been produced. This explains why there is not, as yet, any complete theory. As argued by Paulis [28], difficulties in fully understanding the origin of SL are also due to the fact that the language has a high capacity to transform allochthonous words into Sardinian, bestowing a typical Sardinian habitus upon them. This means that it is often extremely difficult to understand whether a particular type of word was indigenous rather than a transformation of, for example, an Italian or Spanish word.

Generally, as argued by Vona [21] the Paleo-Sardinian language has a pre-Roman or Mediterranean substratum which is still present in modern Sardinian. In particular, according to Sanna [29], it would appear that on one hand, there are some morpho-syntactic elements of clear Semitic origin (spoken by a restricted number of people who came to Sardinia in around 2000 BC) and, on the other hand, some of Indo-European origin.

Pittau [30] has claimed to have found the etymology of many other Latin words in the Etruscan language, after comparison with the Nuragic language. This implies that the Etruscan culture probably exerted a major influence over Sardinian people, who thus were likely to have received many elements from Etruscan that are instead usually considered to be of Latin origin. Pittau [30] has argued that both the Etruscan and Nuragic languages are descended from the Lydian language, therefore both being Indo-European languages, as a consequence of the alleged provenance of Etruscans/Tirrenii from that land (as in Herodotus). Pittau [30] also suggests, as a historical point, that the Tirrenii landed in Sardinia, whereas the Etruscans landed in modern-day Tuscany.

Blasco Ferrer [31] hypothesizes immigration from the ancient Iberian peninsula to Sardinia dating back from the Mesolithic to the entire Neolithic period, with a subsequent derivation of the Paleo-Sardinian language from paleo-Basque and paleo-Iberian languages.

As clearly explained by Wagner [13], the greatest linguistic expert on SL, Sardinia is located in the centre of the Mediterranean sea and, as a natural consequence, it has been influenced by several different cultures from the surrounding areas. More specifically, the Libyan-Sardinian-Iberian front (from south to north) is most connected with the north African toponymy, while the Anatholic/Lydian-Sardinian-Iberian front (from east to west) appear to be closest to the Hellas and Anatolia areas. However, the real origins of the SL remain shrouded in mystery [13].

Whatever the origin of the SL, the scientific community agrees that the sub-regions of Barbagia and Ogliastra represent the areas where the linguistic, ethnic, and genetic specificities are best preserved [19], [32–33]. Aboriginal Sardinians did not mix with the foreigners who primarily conquered the island’s coastal and plain areas [14], [33–34]; quite the contrast, they abandoned those areas to settle in the mountainous inland areas of central Sardinia, and precisely in the areas today called Barbagia and Ogliastra, which remained mainly inaccessible to foreigners [19], [32–34]. As a consequence of the geographical isolation, together with the unavoidable inbreeding due to the small population, a genetic and linguistic drift, both within the island and the rest of Europe, has been scientifically recognized [32], [35–36].

### Ethnopedological approach

Ethnopedological investigations can be conducted in three main ways, *i*.*e*. ethnographic, comparative, and integrated [5], [37].

Very briefly, in the ethnographic approach, the ethnopedological information is not compared with scientific soil information. The comparative approach aims to establish similarities and differences between local knowledge and scientific information, with no consideration for the socio-cultural contexts from which perception, beliefs, cognition, and practices derive. In this research, we took an integrated approach with the main goal being to link soil and land perception and knowledge in order to promote feasible, sustained local endogenous development from an interdisciplinary perspective [6].

The research was conducted in four main stages.

#### Toponyms collection

A variety of data collection strategies was used. Toponyms were collected from the following sources: i) from the Sardinia Geoportal [38], which contains all toponyms belonging to different cartographic sources such as the Regional Technical Maps at 10k, the National Maps (25k, 50k, 100k, 1000k), the Italian Touring Club Map, the DBPrior at 10k, the Cadastral Maps (200k and 400k), the Historical Map of Sardinian Toponyms [39]; ii) from the Municipal Library and State Archives of Sardinia; and iii) from personal narratives recorded during informal interviews conducted with the attenders and/or the owners of the areas characterized by toponyms of unknown meaning.

#### Database creation and toponyms translation

A total of 7742 toponyms were collected. All toponyms were entered into a database comprising twenty-three spreadsheets (one for each municipality of the investigated area) giving a complete list of collected toponyms. Each toponym (as originally reported in the map) was characterized by an identification code, the original cartographic source (name and scale), the geographic position (geo-referenced), the meaning as obtained through translation (obviously only for those toponyms reported in Sardinian language), the bibliographic source of translation, and the assigned category (*vide infra*).

The toponyms were translated using two main strategies. The first approach used some of the most important research conducted to date on Sardinian toponymy [13], [40–43]. This aspect was particularly awkward because, as previously explained, there are often substantial differences between the various theories on the development of SL, meaning that opposite results have sometimes been produced. Indeed, in some cases, different meanings have been attributed to the same toponym by different authors. In order to avoid incongruence in the translation of toponyms, only the most accepted research among etymologists was considered [13], [40–43].

As a further strategy, toponyms of formally unknown meaning, for which an official translation is still lacking, were deciphered and recorded through direct discussions with attenders/owners of the areas of interest, as illustrated previously.

#### Toponyms categorization

Toponyms with a similar prevalent meaning were grouped into the following seven reference categories (C):

1. soil (SC): a meaning that clearly reflects a generic and/or specific soil behaviour such as texture, fertility, colour, and permeability;
2. soil/geology (SGC): a meaning directly and/or indirectly connected with geological and pedo-geological features such as soil stoniness, type of rock, and their prevalent colour;
3. soil/morphology (SMC): a meaning connected to morphology and pedo-morphological features such as the form of the relief, the soil position along the relief, the kind of morphology, and the appearance;
4. morphology/vegetation (MVC): a meaning describing a connection between morphology and vegetation such as, just to mention one of the most common cases, the type of plant species and their location in the relief;
5. morphology/fauna (MFC): a meaning with a clear reference to morphology and fauna such as the kind of animal and their location along the relief;
6. vegetation (VC): a meaning having a direct reference with the vegetation and/or simply to a peculiar kind of plant species;
7. soil cover/land uses (SLC): a meaning describing a connection between soil cover, vegetation, and land uses such as the type of soil use and the main kind of plant species used for such purpose.

Obviously, these categories are a massive simplification but one which was necessary indeed. The meaning of some toponyms was not always related to just one feature and/or category, but rather can represent a multiple meaning thus falling at the edge of two or more of the recognized categories. In such cases, the category was assigned according to the prevalent meaning.

Moreover, many other toponyms do not belong to any of these categories, such as those in Italian or, generally speaking, those in Italian and SL identifying the names of a water course, the name of the landowner (often reported as the family name), the name of the streets, the building, mines, and the infrastructures (such as schools, main roads, airports, pipelines, and railway stations). Such toponyms were simply identified as belonging to other toponyms in SL not otherwise classified (OTSL) or as toponyms in Italian (OTI).

Finally, toponyms that are still of unknown meaning and/or obscure origin were classed as “untranslatable” (UT).

#### Data processing

All data collected by categorization, as well as all cartographic elaborations (distribution and diversity of toponyms) were managed using the compiled database coupled with a geographical information system (GIS). The database was implemented with the addition of the soil map of Ogliastra [44], also in order to draw comparisons between local soil knowledge (deducible from toponyms meaning) and scientific information provided by the soil map. Additionally, with the information provided by the soil map, a pedodiversity index, according to the Shannon's entropy or diversity index, was prepared as following reported [45]:

$$H=-\sum_{i=1}^{S}pix\ln pi$$ (1)

where p_i_ is the proportional abundance of class i and s is the number of soil units.

Using the same formula, a topodiversity index was also prepared (in this case s represents the number of toponymy units).

An overall correlation between pedodiversity, topodiversity, altitude, municipality surface, and toponym categories was performed by means of principal factor analysis (PFA). Principal factors were rotated using the varimax method, thereby maximizing variance distances between groups.

## Results and Discussion

### Toponym collection, translation, and categorization

Generally speaking, the investigated traced etymologies and the linguistic links yield very interesting languages patterns (S2 Table), ranging from Asian to Mediterranean regions, including Northern Europe. Clearly, several words (S2 Table) are linked to Latin. However, this language had already spread prior to the expansion of Roman empire. On the other hand, as explained later, the etymon of several words still remains unknown today. As was to be expected, most of toponyms refer to environmental features, satisfying, as will be explained more fully, the primary needs of mankind and possibly representing the relict of the old linguistic substratum, foregoing the later fluxes of foreign peoples and idioms.

Table 1 summarises the results of the collection, translation, and categorization of the toponyms.

**Table 1:** Categorization of Ogliastra toponyms.

Municipality	Toponyms in Sardinian language									Toponyms in Italian	Toponyms per municipality	Area (kmq)	Elevation (m a.s.l.)
	Environmental categories							Others					
	SC^a^	SMC^b^	SGC^c^	MFC^d^	MVC^e^	VC^f^	SLC^g^	OTSL^h^	UT^i^	OTI^k^			
Arzana	28	65	14	19	51	43	5	175	181	127	708	162,4	672
Barisardo	0	16	10	0	4	18	3	27	22	64	164	37,5	51
Baunei	16	133	20	35	68	33	4	254	288	57	908	212,1	480
Cardedu	1	5	7	1	4	5	2	22	14	90	151	31,9	40
Elini	2	5	2	0	0	8	0	16	7	16	56	10,6	472
Gairo	8	20	22	6	40	29	2	76	47	65	315	78,8	685
Girasole	1	3	2	1	1	4	1	10	4	44	71	13,2	8
Ilbono	4	7	6	0	6	16	0	42	39	43	163	31,1	400
Jerzu	17	28	12	14	23	43	5	117	38	108	405	102,6	427
Lanusei	3	14	5	3	6	28	2	78	28	76	243	52,6	595
Loceri	0	11	5	0	3	12	0	20	7	27	85	20,9	190
Lotzorai	5	3	2	1	2	5	2	11	2	38	71	16,5	11
Osini	5	10	3	5	11	15	5	47	15	57	173	39,6	645
Perdasdefogu	10	31	15	0	10	28	7	119	18	59	297	77,1	599
Seui	6	30	26	16	39	50	8	254	111	80	620	148,1	820
Talana	18	34	19	8	42	48	9	136	99	64	477	118,0	682
Tertenia	6	31	9	16	18	42	3	147	41	140	453	116,7	121
Tortolì	1	7	8	1	8	16	6	52	17	133	249	40,5	13
Triei	2	13	8	4	7	13	1	28	16	42	134	32,9	140
Ulassai	25	44	22	10	23	27	14	130	93	61	449	123,3	775
Ussassai	7	7	16	1	12	23	4	62	47	34	213	47,6	511
Urzulei	29	45	30	9	27	36	12	110	164	42	504	131,5	710
Villagrande	29	88	34	22	70	98	13	218	107	154	833	210,9	700
**Toponyms for category**	223	650	297	172	475	640	108	2151	1405	1621	7742	1856,6	

Reference Categories (C):

^a^SC = soil.

^b^SMC = soil/morphology.

^c^SGC = soil/geology.

^d^MFC = morphology/fauna.

^e^MVC = morphology/vegetation.

^f^VC = vegetation.

^g^SLC = soil cover/land uses.

^h^OTSL = other toponyms in Sardinian language.

^i^UT = untranslatable.

^k^OTI = other toponyms in Italian.

**Fig 2:**
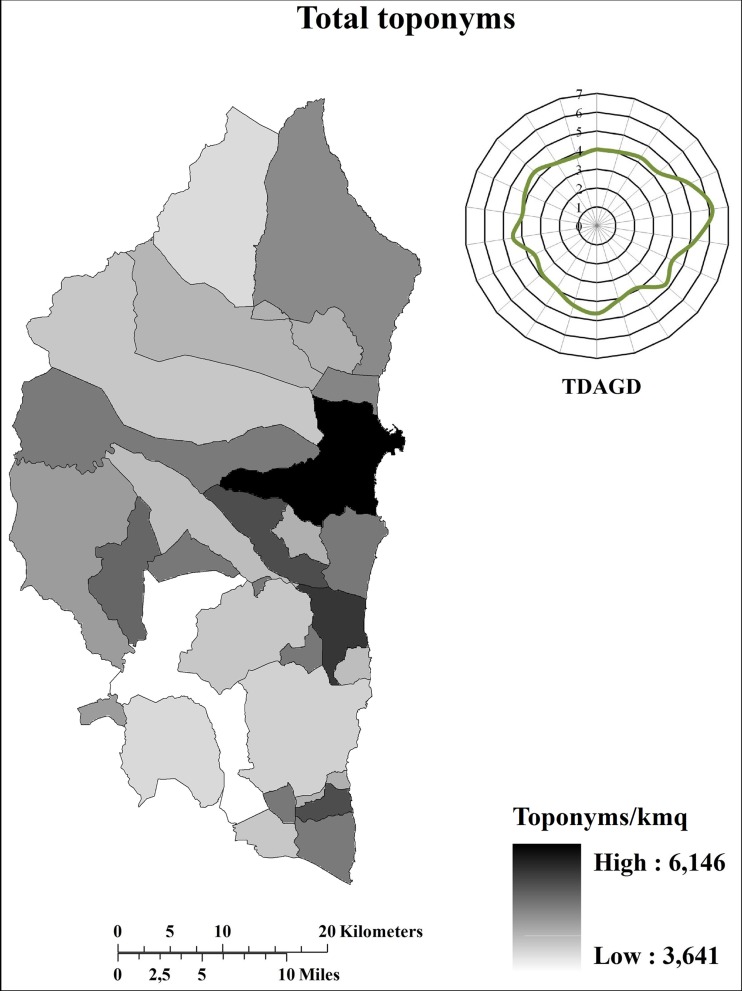
Distribution (toponyms/km^2^) of total toponyms. TDAGD = Toponym distribution (toponyms/km^2^) along the geographical directions. The graphic representation of the TDAGD was achieved by positioning the centre of the graphic over the municipality of Elini, representing the geographic centre of Ogliastra.

**Fig 3:**
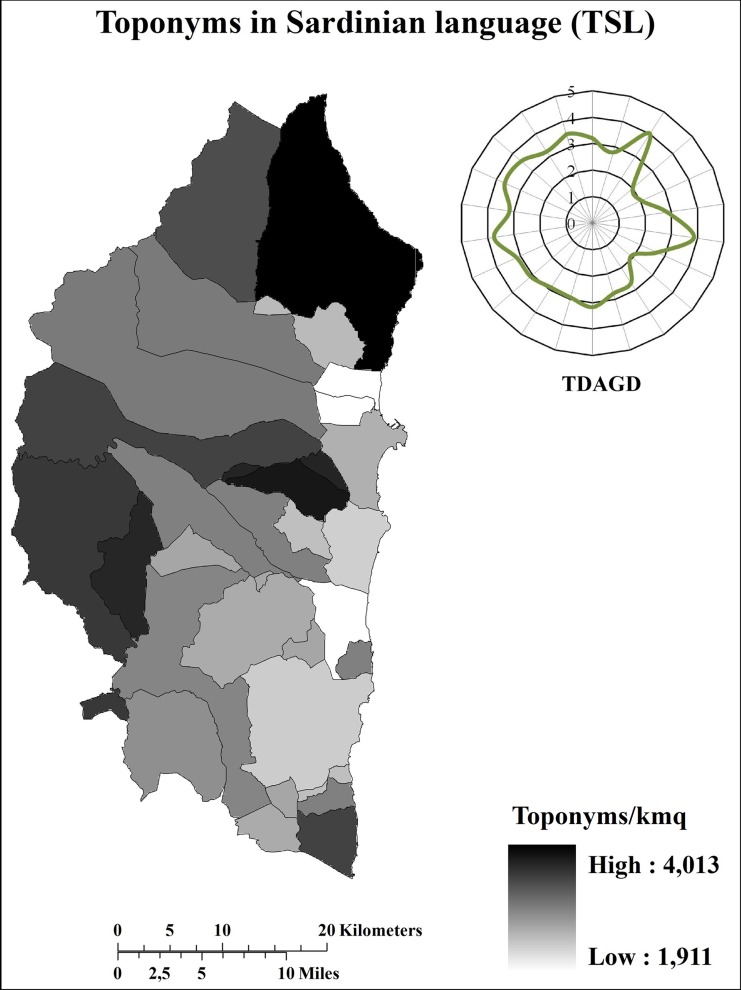
Distribution (toponyms/km^2^) of toponyms in Sardinian language. TDAGD = Toponym distribution (toponyms/km^2^) along the geographical directions. The graphic representation of the TDAGD was achieved by positioning the centre of the graphic over the municipality of Elini, representing the geographic centre of Ogliastra.

**Fig 4:**
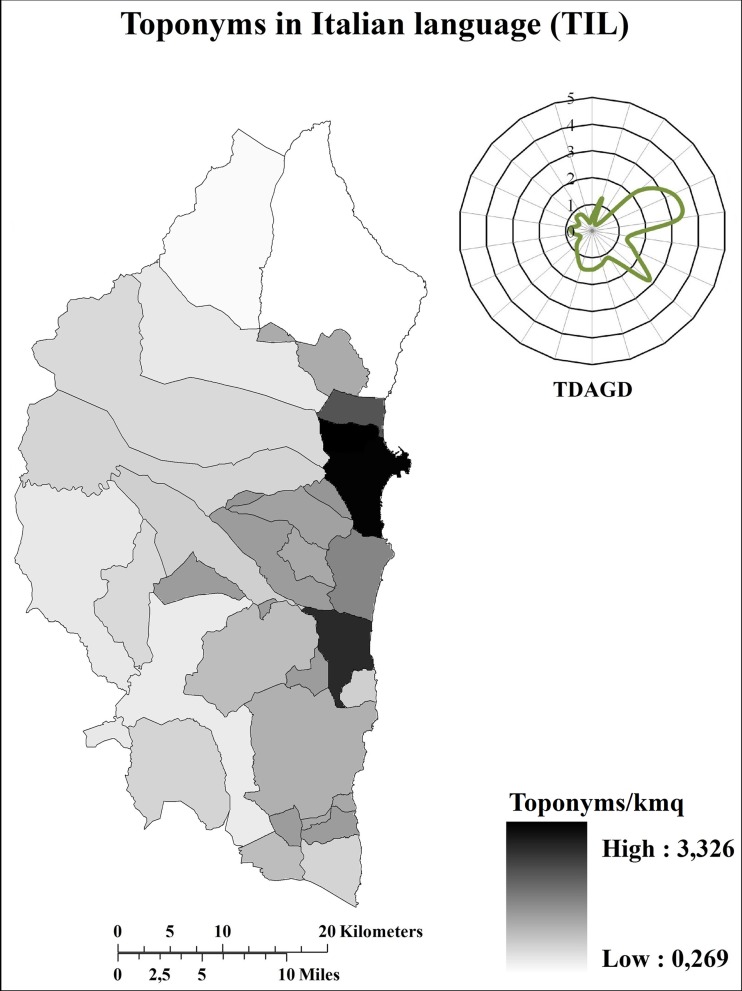
Distribution (toponyms/km^2^) of toponyms in Italian. TDAGD = Toponym distribution (toponyms/km^2^) along the geographical directions. The graphic representation of the TDAGD was achieved by positioning the centre of the graphic over the municipality of Elini, representing the geographic centre of Ogliastra.

On the whole, the Ogliastra sub-region was characterized by more than 7700 toponyms (4 toponyms/km^2^) 79% of which were in SL, whereas just 21% were in Italian (IL). In order to avoid the influence of the area dimension that varies considerably from one municipality to another (Table 1), toponym distribution was analysed in terms of density (Figs. 2–4).

It is immediately clear that the distribution differs tremendously, both in terms of quantity and quality, between inland and coastal areas. In terms of quantity, total toponym density is greater along the coast than in the inland. Indeed, total toponym density (Fig. 2) ranges between 4–5 toponyms/km^2^ along the inner part of the sub-region, yet exceeds 6 toponyms/km^2^ along the eastern coastal area. In terms of quality, toponyms in SL were more widespread in the inland areas than along the coasts (Fig. 3), whilst the opposite is true for toponyms in IL (Fig. 4). Both quantitative and qualitative distribution patterns appear to be closely connected with the historical vicissitudes that characterized the investigated area. Certainly, as briefly described in S1 Table, all the invaders that succeeded each other in the sub-region were able to conquer mainly the coastal areas, whereas the inner part of the sub-region remained under the dominion of the indigenous community. Consequently, there was increasing fragmentation of the coastal territory with a subsequent conation of new toponyms in order to identify the presence of new areas, buildings, and infrastructures realized from the new civilization. By contrast, the inner part of the sub-region remained preserved from such colonization and the subsequent environmental fragmentation. This means that during the same period, the toponymy heritage characterizing the inner areas remained unaltered both in terms of quality and quantity. Conversely, along the coastal areas, the toponymy heritage was heavily influenced, not only in terms of quantity but also in term of quality, since the autochthonous language was progressively lost and/or changed in favour of the language(s) of the conqueror(s). It was the Pisans and Genoese (1215 AD-1326 AD) who first embarked upon an intensive campaign of “Italianization”, which was definitively resumed in the 18^th^ century under the dominion of the House of Savoy, primarily affecting the coastal areas (S1 Table). In contrast, the mountainous interior areas of Barbagia and Ogliastra remained linguistically and culturally isolated and strongly averse to any such changes [21].

On the toponyms in SL, 77% were characterized by a well-recognized etymological meaning, while for the remaining 23% the meaning remains obscure. Of the toponyms in SL with a recognized etymological meaning, the largest proportion (54%) was characterized by a meaning directly or indirectly connected to specific environmental features, such as those reported in the seven recognized categories (Table 1). Such a high percentage of toponyms connected to environmental features clearly shows that the inhabitants of the Ogliastra sub-region had historically named sites mainly according to the characteristics of their surrounding environment (S2 Table). More specifically, the environmental categories with the highest quantity of toponyms (Table 1) were the soil/morphology category (SMC) and the vegetation category (VC). Relief [46] and vegetation are some of the most important and recognisable environmental features characterizing the whole of Ogliastra [44]. Indeed, this territory has an extremely complex and articulated/irregular geomorphology, mainly consisting of a Palaeozoic metamorphic and igneous rock basement with an overlying Mesozoic calcareous formation [47], where small plains (from 0 to 300 m a.s.l.), hilly areas (from 300 to 700 m a.s.l.), calcareous-dolomitic plateaus (till to 1300 m a.s.l.), and mountain chains (over 700 m a.s.l.) are often concentrated in just a few square kilometres [27]. An analysis of acclivity [27] clearly shows that 66% of the territory is characterized by slopes > 20% and that the most widespread acclivity classes (covering around 31% of Ogliastra’s surface area) are those with slopes between 30% and 50%.

The importance and specificity of the Ogliastra landscape has been also demonstrated by the following important aspects based on the presence of: i) Natural Monuments instituted by regional laws [46]; ii) a specific proposal [46] for the institution of a Geomorphologic Reserve aiming to preserve the calcareous-dolomitic plateaus (commonly referred by the local populations as *Tacchi* or *Tonneri*, meaning “heel”, due to their specific shape); iii) several national and international scientific research undertakings about the Ogliastra geomorphology [46], [48–52]; and iv) the presence [46] of important Geosites or Geomorphosites, *i*.*e*. landforms with a specific shape that alone or in connection with other bioecological or anthropic elements, can become objects of heritage [53]. Consequently, the shape of the relief, constituted by many long-distance recognizable morphological elements, had historically represented an important visual point of reference for the Ogliastra inhabitants [43]. For all these reasons, many toponyms make specific reference to pedo-morphological aspects.

In terms of vegetation, the Ogliastra sub-region was historically characterized by the presence of extended Mediterranean woody formations, mainly belonging to the thermo-mesomediterranean holm oak (*Quercus ilex* L.) and cork oak (*Quercus suber* L.) series [27]. However, such potential series are often substituted, along with changing of pedo-environmental conditions, by different series with a consequent creation of an extremely fragmented and articulated vegetational landscape [27]. This means that vegetation, together with the shape of the relief, represents the most recognizable environmental elements of the Ogliastra landscape. As expected, such elements are often recognizable at a glance, *i*.*e*. from a very long distance, since they were formerly utilized as specific geographic reference points. Indeed, such a function attributed to specific environmental elements and consequently to their toponyms, was particularly important for an area where an extremely complex morphology makes the orientation along the space an unsolvable problem if specific environmental references are not available. For such reasons, there is an ancient tradition in naming a place according to specific attributes connected to a peculiar shape of the relief and/or the presence of a particular kind of vegetation both in terms of species or vegetal associations. Such an ancient tradition has been demonstrated by the fact that such types of toponyms are mainly of pre-Latin origin [13], representing some of the oldest historic evidence of the ancient Sardinian language [13], [43].

### Toponyms and soil features

**Fig 5:**
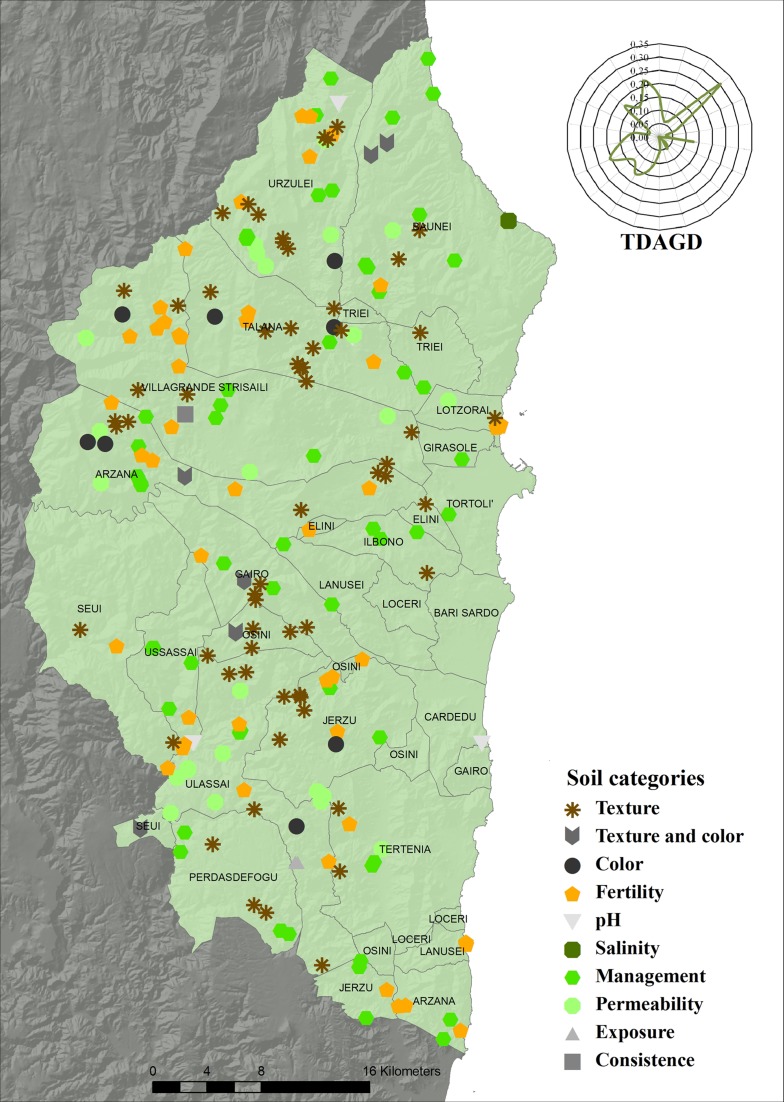
Distribution of soil toponyms. TDAGD = Toponym distribution (toponyms/km^2^) along the geographical directions. The graphic representation of the TDAGD was achieved by positioning the centre of the graphic over the municipality of Elini, representing the geographic centre of Ogliastra.

The specific conformation of Ogliastra probably also represents the main explanation underlying the reasons for soil toponym types (Tables 2 and 3) and distribution (Fig. 5).

**Table 2:** Categories and sub-categories of soil toponyms and their correspondence with prevalent soils.

Prevalent soils		Texture^a^			Texture and color^b^		Color^c^			Fertility^d^			SA^e^	MA^f^		LP^g^	pH^h^		EX^i^	SC^k^	Total for prevalent soil
		G	S	C	WC	RC	W	R	B	H	G	EL		CU	PA		AC	AL			
Typic Palexeralfs and Typic Haploxeralfs		0	1	21	0	3	0	2	1	0	0	1	0	0	2	15	2	0	0	0	48
Typic, Dystric and Lithic Xerorthents with Dystric Typic and Lithic Xerochrepts		2	7	12	0	0	1	0	0	9	1	2	0	37	14	1	0	1	2	0	89
Lithic and Typic Rhodoxeralfs withTypic Haploxerolls		0	0	9	2	2	0	0	0	1	0	0	1	4	0	1	0	0	0	0	20
Lithic Xerorthents		1	3	0	0	0	4	0	0	0	0	8	0	0	3	6	0	1	0	0	26
Typic, Vertic, Aquic and Mollic Xerofluvents		0	8	0	0	0	0	0	0	13	0	0	0	0	0	2	0	0	0	0	23
Typic Xerumbrepts		0	0	0	0	0	0	0	0	9	4	0	0	0	0	0	0	0	0	1	14
Typic and Aquic Xeropsamments with Typic Fluvaquents		0	0	0	0	0	0	0	0	3	0	0	0	0	0	0	0	0	0	0	3
**Total for soil toponyms sub-categories**	n	3	19	42	2	5	5	2	1	35	5	11	1	41	19	25	2	2	2	1	223
	%	1,3	8,5	18,8	0,9	2,2	2,2	0,9	0,4	15,7	2,2	4,9	0,4	18,4	8,5	11,2	0,9	0,9	0,9	0,4	

^a^Texture: G = gravelly, S = sandy, C = clayey.

^b^Texture and color: WC = white clay, RC = red clay.

^c^Color: W = white, R = red, B = black.

^d^Fertility: H = high, G = good, EL = extremely low.

^e^Salinity.

^f^Management: CU = cultivation, PA = pasture.

^g^LP = Low permeability.

^h^pH: AC = acid, AL = alkaline.

^i^EX = Exposure.

^k^SC = Soft consistence.

**Table 3:** Comparison between some selected soil toponyms (indigenous knowledge) and the corresponding prevalent soils (scientific information) according to the area soil map.

Toponym in Sardinian language	Soil category and sub-category	Simplified linguistic root	Translation and meaning	Prevalent soils
Accu lassinosu	Texture and color; Red clay	*Baccu (La)*: valley; *Lapsare (La)*: slip	A slippery valley due to the presence of red clay [10]	Typic Palexeralfs and Typic Haploxeralfs
Bacu su ludas	Texture; Clayey	*Baccu (La)*: valley; *Lutum (La)*: mud	Valley of mud [40]	Typic Palexeralfs and Typic Haploxeralfs
S'orgosa manna	Low permeability	*Orgosa (Pr)*: wetland, swamp*; Magna (La)*: greater	A large valley characterized by temporary pond [40]	Lithic Xerorthents
Tulargius	Fertility; High	*Tula*, *Tabula (La)*: seedbed, flowerbed	An area that can be sown in just one day [40]	Typic, Dystric and Lithic Xerorthents with Dystric Typic and Lithic Xerochrepts
Mainesa	Texture; Clayey	*Maina (La)*: clayey	A clayey area [40]	Typic Palexeralfs and Typic Haploxeralfs
Ludu	Texture; Clayey	*Lutum (La)*: mud	The mud [10]	Lithic and Typic Rhodoxeralfs withTypic Haploxerolls
Nessicuru	Fertility; Extremely low	*Nesigu*, *mesigu*, *mezzo(It)*: rotten	Soils without any value [43]	Lithic Xerorthents
Serra terralba	Color; White	*Serra (La)*: ridge; *Terra (La)*: soil; *Alba (La)*: white	The ridge of the white soils [42]	Typic, Dystric and Lithic Xerorthents with Dystric Typic and Lithic Xerochrepts
Solanas	Exposure	*Solanus (La)*: sunny	Sunny soils [42]	Typic, Dystric and Lithic Xerorthents with Dystric Typic and Lithic Xerochrepts
Martana	Soft consistence	Unknown	Mushy soil [42]	Typic Xerumbrepts
S'aspro	Fertility; Extremely low	*Asper (La)*: rugged	A rugged area with unfertile soils [42]	Lithic Xerorthents
Settile	Management; Cultivation	*Séttile (Pr)*: small hill	A flat surface with soils suitable for cultivation [42]	Typic, Dystric and Lithic Xerorthents with Dystric Typic and Lithic Xerochrepts

It = italian.

Pr = pre-roman, proto-sardinian.

La = latine (Roman origin).

On 223 toponyms connected to soil behaviour (Table 2), 29% describe attributes regarding soil texture (gravelly, sandy, or clayey), 27% are connected to soil management (cultivation and pasture), 23% are toponyms with a more or less strict reference to soil fertility (high, good, and extremely low), and 11% clearly refer to low soil permeability. All these categories and their respective sub-categories, represent soil attributes that were easy to describe for users interested in soil practical uses such as grazing and agriculture. This is why soil toponyms were coined in order to immediately describe a few extremely important aspects of soil attributes representing possibilities (high and good fertility) or obstacles to the exploitation of the soil resource (Table 3). There is clearly a greater presence of soil toponyms describing soil limitations rather than soil qualities (Table 2). Intuitively, there was an extreme need to immediately recognize these areas that, due to their significant soil limitations, were unable to be used for human daily needs. This hypothesis is also borne out in considering the extremely complex conformation of Ogliastra. Indeed, the grounded prospect of covering huge distances in a morphologically articulated territory with the final perspective represented by an area characterized by important soil limitations, was probably one of the key factors in defining those areas by toponyms connected with soil attributes linked to important limitations for practical everyday uses. The remaining 10% of soil toponyms (Table 2), even if representing a clear indication of the intimate indigenous knowledge of the landscape, are linked to soil properties generally more difficult to describe (soil salinity, soil reaction, and soil consistency), or less clearly connected to soil uses for human needs (soil texture/colour, soil colour, and soil appearance). The fact that morphology had an important influence on soil toponyms characteristics is also clear from the way soil toponyms are distributed throughout the territory (Fig. 5). Indeed, only 4.5% of soil toponyms (ST) are located along the coast, whereas the distribution of ST is clearly abundant in the inland areas, especially heading south-west and north-west, *i*.*e*. towards the hilly and mountainous areas of Barbagia and Ogliastra.

The localization of areas characterized by peculiar kind of soil attributes was probably crucial in order to achieve a better utilization of the surrounding extreme environment. By contrast, there was probably no particular reason to localize, through specific soil toponyms, a suitable area along the coast (especially for the plains), since such areas were far easier to reach. From this point of view, the use of soil toponyms was historically intended to meet the community needs in terms of making better use of environmental resources.

#### Indigenous and scientific knowledge of soil resources

Tables 2 and 3 clearly show that indigenous knowledge of soil resources was highly comparable with modern scientific knowledge. The indigenous knowledge represented by the meaning of soil toponyms is effectively pretty consistent with the scientific information provided by the area’s soil map, *e*.*g*. the taxonomic allocation of the prevalent soils at the Subgroup level [54]. For example, 71% of soil toponyms with a prevalent meaning identifying a clayey soil texture fall within areas characterized by Alfisols, *i*.*e*. soils with an argillic horizon. Soil toponyms with a meaning indicating the presence of red clays are often located in areas characterized by Lithic and Typic Rhodoxeralfs, *i*.*e*. Alfisols with a red argillic horizon. In terms of soil fertility, areas delimitated by toponyms indicating highly fertile soils are prevalently characterized by Xerofluvents, Xerumbrepts, and Haploxerolls. Conversely, areas characterized by infertile soils, such as Lithic Xerorthents, were clearly marked off by soil toponyms indicating this important limitation. Clearly, this is not surprising in conceptual terms, however such a high correlation between indigenous and scientific knowledge clearly shows that the ancient peoples of the sub-region were very intimate with their territory, showing an in-depth understanding of the environmental resources without any specific formal competence. In examining the toponyms indicating some aspects of soil management, a seeming contradiction with the previous toponyms on soil fertility immediately becomes clear. Even if the ancient populations had a specific familiarity with highly fertile soil locations, agricultural activities were mainly located in areas featuring less productive soils such as Xerorthents and Xerochrepts. However, this contradiction is merely apparent, since areas characterized by more fertile soils were often located in constraining morphological conditions, or characterized by a reduced spatial extension. On the other hand, highly fertile soils often fell into areas of temporary ponds, *i*.*e*. small and shallow water bodies characterized by alternating phases of drought and flooding. The high presence of toponyms (11%) indicating low soil permeability was linked to the presence of such ecosystems, that extensively characterize the Ogliastra sub-region, due to the presence of favourable geologic (impermeable underlying rock), morphologic (undulating topography), and pedologic (clay-enriched subsurface horizons or the prevalence of a lithic contact) conditions [55]. In ancient times, such sites were left in their original state and human activities located far from their borders, due to the presence of environmental conditions conducive to malaria. For all these reasons, agricultural and pastoral activities were prevalently based in territories with favourable morphological conditions, larger spatial extension, and safer environmental conditions, even if with potentially less fertile soils. In point of fact, in their *ante litteram* Land Evaluation criteria, ancient Sardinian people considered that soil fertility alone was much less relevant than the overall agricultural feasibility of a land, including geomorphology, extension, and environmental safety against enemies and diseases.

### Topodiversity and pedodiversity

The PFA results relating to pedodiversity, topodiversity, altitude, municipality surface, and toponym categories are shown in Table 4.

**Table 4:** Factor loadings of a factor analysis (n = 23).

	Factors	
Parameters	F1	F2
SC	0,567	0,664
SMC	**0.912**	0,240
SGC	0,559	0,695
MFC	**0.942**	0,178
MVC	**0.860**	0,432
VC	0,658	0,571
SLC	0,394	0,673
Total toponyms	**0.917**	0,376
Municipality surface	**0.846**	0,402
Altitude	0,266	**0.803**
Pedodiversity	0,197	**0.911**
Topodiversity	0,209	**0.812**
Variance (%)	44.9	36.8
Eigenvalues	5,391	4,415

Extraction Method: principal factor analysis (PFA); Rotation Method: Varimax; bold loadings > 0.7.

SC = soil.

SMC = soil/morphology.

SGC = soil/geology.

MFC = morphology/fauna.

MVC = morphology/vegetation.

VC = vegetation.

SLC = soil cover/land uses.

Such a multivariate statistical analysis confirms that morphology was the main environmental factor underlying the presence, typology, and distribution of the investigated toponyms. Two factors were extracted through PFA, both related to morphological aspect. Factor 1, accounting for 44.9% of total variance, links all morphology-related toponyms (soil/morphology, morphology/vegetation, and morphology/fauna categories) positively to total toponyms and municipality surface. Therefore, Factor 1 can be interpreted as the “influence of municipality surface on toponym abundance”. This factor simply shows that by increasing municipality surface, a total increase of total toponyms was expected, with particular reference to those connected to morphological features. Factor 2, explaining about 36.8% of the variance, extracts altitude, pedodiversity, and topodiversity, all positively concordant. Thus, this factor can be interpreted as “the influence of morphology on pedodiversity and topodiversity”. Such a factor clearly implies that both pedodiversity and topodiversity increase with altitude. As previously explained, Ogliastra is characterized by an exceptionally multifaceted/irregular morphology. This complexity is particularly enhanced along hilly areas and mountain chains, which are characterized by a huge variability of morphology, often featuring different gradient slopes, substrates, and vegetation cover. Such an extreme environmental fragmentation obviously results in different pedo-environmental conditions, with the consequent formation of peculiar/specific kinds of soil for each environment. As a whole, such conditions end up being reflected in greater pedodiversity.

**Fig 6:**
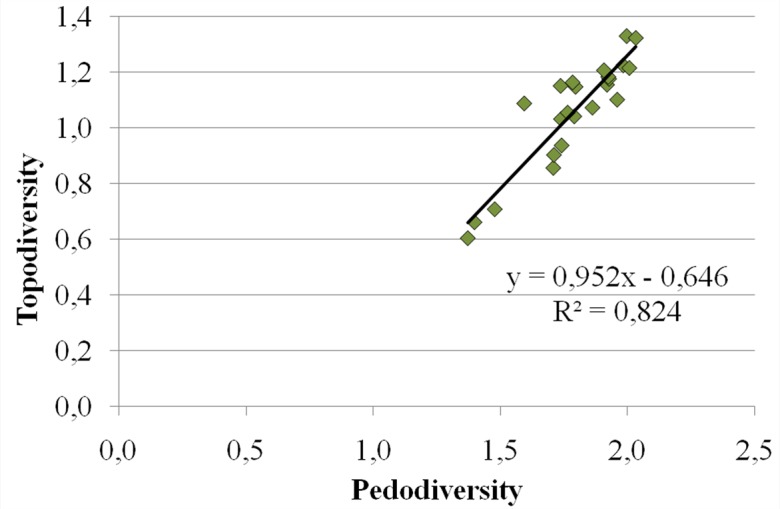
Least squares regression of pedodiversity vs. topodiversity.

For these reasons, such environments are characterized by different kinds of toponyms, due to the need, in ancient times, to immediately recognize different parts of the territory characterized by different environmental features. From this point of view, the least squares regression analysis (Fig. 6) of pedodiversity vs. topodiversity shows a strictly close distribution, with a highly significant correlation index (R^2^ = 0.824).

This confirms that, even if only indirectly, pedodiversity and topodiversity are consistently linked each other. Thanks to the PFA analysis and in agreement with the previously presented results, it would appear that such a connection could be morphologically based.

## Conclusions

This study took an integrated ethnopedological approach with a view to investigating the meaning and distribution of the toponyms used in traditional and recent cartography of Sardinia (southern Italy).

Morphology appears to be the main environmental factor explaining the presence, distribution, and typology of the investigated toponyms. In particular, a highly significant correlation among the number and meaning of toponyms, altitude, pedodiversity, and topodiversity was ascertained, highlighting the fact that such a connection may be morphologically based.

From a purely linguistic point of view, several local languages, now used by small ethnic groups, are likely to vanish during the twenty-first century. This highlights the magnitude of the effort needed to compile an inventory of and to analyse the specific forms of indigenous perception, familiarity, and management of the soil and land resources before they disappear entirely. Clearly, such disappearances are particularly enhanced in conditions where indigenous expertise is no longer transmitted (both as oral or written information) over the generations.

Overall, the research shows that an integrated ethnopedological approach, able to fully combine ancient indigenous and modern scientific knowledge, could be of great interest and value in hindering the massive loss of indigenous knowledge. For these reasons, ethnopedological investigations may represent one of the possible approaches by which to best preserve such indigenous knowledge.

## Supporting Information

S1 Table A schematic overview of the main historical events of Sardinia. (DOC)

S2 Table Etymology and linguistic links of selected toponyms in Sardinian language. (DOC)
