# Supplementary material for: Ethnopedology in the Study of Toponyms Connected to the Indigenous Knowledge on Soil Resource
Source: PLoS One. 2015 Mar 19;10(3):e0120240. doi: 10.1371/journal.pone.0120240 (PMC4366272; doi:10.1371/journal.pone.0120240)
Supplement: S1 Table — (DOC) [file pone.0120240.s001.doc]

**S1 Table. A schematic overview** of the main historical events of Sardinia.

| **Period** | **Age** | **Main aspects** | **Additional information** |
| --- | --- | --- | --- |
| Lower Paleolithic | 450–120k BC | Earliest finds of the presence of hominins, who probably came from the Italian peninsula and specifically from Etruria, perhaps via the island of Corsica | Genetic and population genetics studies have shown that Sardinians are an ethnically distinct and homogenous group [33-34] representing one of the most ancient populations of Europe [34], [56] with origins lie in an early split in the Caucasoid group [33] with a particular genetic make-up that differs from that of other Caucasians and even mainland Italians [18], [56]. This is mainly due to genetic drift, though other reasons, such as relations with pre-Indo-European Neolithic peoples may also have contributed to this distance. |
| Neolithic | 6000 BC | First permanent settlements | Sardinia was inhabited by the Nuragic Sardinians, also called Ioalei or Ilienses, a population of presumed Libyan origins [13]. |
| Early Bronze Age | 1800–1600 BC | A culturally and technologically advanced culture (Nuragic Civilization), had its origins | The Nuragic Civilization built the *Nuraghi*, *i.e.* fortified stone towers mainly intended for defence and habitation [21], [56]. They were amongst the most important metal producers in Europe [57-58] with major commercial exchanges involving several populations [21] both in the Bronze Age (Balearic Islands, Corsica, France, the Iberian Peninsula, Mycenae and Cyprus) as well as in the Iron Age (Phoenicians and Etruscans). |
| Late Bronze Age | 1500–1300 BC | Nuragic Sardinians spread in the Mediterranean Sea | For some author [59] they were known by Egyptians as the “Sea People”, also called “Shardana”, or Sherden. |
| Final Bronze Age and Iron Age | 1000 BC | Phoenicians began visiting Sardinia |  |
|  | 509 BC | Phoenicians conquered the coastal areas | A war broke out between the native Nuragic people and the Phoenician settlers. The settlers called for help from Carthage and the island became a province in the Carthaginian Empire. However, the central mountainous land (Barbagia and Ogliastra) remained under the rule of the Nuragic Sardinians [57-58]. |
| The Roman Empire and the Vandalic invasions | 238 BC–455 AD; 455–534 AD | The Roman and Vandalic invasions represented the end of the Nuragic civilization | The end of the Nuragic Civilization has been dated by archaeologists and historians to 476 AD with the “official” beginning of Middle Ages. This means that Nuragic Civilization would not only have partially survived the Roman invasion, but in part also the Vandalic conquest. The Roman domination brought Latin to Sardinia, which from this historical moment on was to represent the main linguistic base of the SL. However, Latin was not able to completely supplant the Paleo-Sardinian language and some obscure roots remained unaltered [13]. This was particularly true for those Nuragic populations located in the inner part of the island, such as Barbagia and Ogliastra. |
| The Byzantine Empire and the Mediaeval period | 534–900 AD; From 900 AD | Sardinia was characterized by an administrative form called *Giudicato* (derived from the Byzantine magistrate title of *“iuidici”*, literally judge, or magistrate) | The Sardinian language (SL) developed in its own way and was fairly homogenous and compact [13] with only two major varieties of Sardinian, *Campidanese* (south) and *Logudorese* (north). This is why these are considered the archetype of all modern varieties of Sardinian [21]. |
| The first Italian influence | 1215–1326 AD | Pisans and Genoese dominion | The linguistic frame was strongly influenced by Pisans and Genoese, who embarked upon an intensive campaign of “Italianization”, primarily affecting the coastal and plain areas of Sardinia, whereas Barbagia and Ogliastra continued to remain linguistically and culturally isolated and averse to the linguistic innovation [21]. |
| The Spanish period | From 1323 AD | Through an intensive military campaign Sardinia was occupied by the Crown of Aragon | The Crown of Aragon began with an impressive process of acculturation that profoundly modified the pre-existing social, cultural, and linguistic make-up of Sardinia [13]. In this way, for the first time, a real situation of diglossia was created [13], where there was an overlap between the language of the conqueror and the language of the conquered [21]. |
|  | From 1479 to 1720 AD | As a result of the personal union of Ferran II of Aragon and Isabel of Castile (the so-called Catholic king and queen) ten years previous, Sardinia fell under Spanish dominion | The island remained under the Iberian dominion for four hundred years, assimilating a number of their traditions, customs, linguistic expressions, and lifestyles. The acculturation process carried out by the Catalans (Crown of Aragon) was so intense, that it was difficult to uproot the Catalan language and culture. In actual fact, strong traces of that language can still be found in modern north-western Sardinian [21]. |
| From the political unification to contemporary era | In 1718 AD | Sardinia became an independent vassal kingdom under the House of Savoy, rulers of Piedmont |  |
|  | In 1861 AD | Sardinia became a region of Italy | At that time, Sardinian was in a state of great language fragmentation [21], an atypical situation for Romance languages [13] which are generally characterized by a relative homogeneity [60]. From the political unification of Italy onwards, there was an ongoing process of Italianization and acculturation underway in Sardinia. |
|  | In 1946 AD; In 1948 AD | Italy became a republic; Sardinia was recognized as an administrative autonomous region supported by a special statute | The Italianization process proceed and progressively brought the SL into disuse in several areas [21]. Consequently, some social classes, with particular reference to the middle and more privileged classes, abandoned the use of SL completely [21]. In addition, the state of great language fragmentation of Sardinian and the sometimes vast linguistic differences between the various Sardinian varieties facilitated the diffusion of Italian which is now used as a kind of “lingua franca”, *viz.* a vehicular language to communicate with the different dialectal communities [13]. |

**References**

1. Contu D, Morelli L, Santoni F, Foster JW, Francalacci P, Cucca F. Y-chromosome based evidence for pre-neolithic origin of genetically homogeneous but diverse Sardinian population: inference for associations scans. PLoS One. 2008;3(1): e1430.
2. Lilliu G. La civiltà dei sardi dal neolitico all’età dei nuraghi. Torino: ERI Edition; 1967.
3. Lilliu G. La civiltà nuragica. Sassari: Delfino; 1982.
4. Montalbano P. Shrdn. Signori del mare e del metallo. Nuoro: Zenia editrice; 2009.
5. Bolognesi R, Heeringa W. Sardegna fra tante lingue. Il contatto linguistico in Sardegna dal Medioevo a oggi. Cagliari: Condaghes; 2005.
