# Supplementary material for: Ethnopedology in the Study of Toponyms Connected to the Indigenous Knowledge on Soil Resource
Source: PLoS One. 2015 Mar 19;10(3):e0120240. doi: 10.1371/journal.pone.0120240 (PMC4366272; doi:10.1371/journal.pone.0120240)
Supplement: S2 Table — (DOC) [file pone.0120240.s002.doc]

**S2 Table. Etymology and linguistic links of selected toponyms in Sardinian language.**

| **No** | **Municipality** | **Toponym in Sardinian language^a^** | **Simplified phonetic transcription^b^** | **Translation^c^** |
| --- | --- | --- | --- | --- |
| **Soil/morphology category** | | | | |
| 1 | Arzana | Accu mundau | *“àc(c)u”* ≡ *“bàc(c)u”*; cfr. <Βάκχος> {Bàkchos} [Gr.] and <Bacchus> [LAT.], both linked to the name of the Thracian-Phrygian deity of wine [61]; by extension, the SL root <BAKKO> also signifies the wine bowl, as well as a geomorphological hollow [42] --- *“mùndau”*; cfr. <mundus> [LAT.] = clean, swept [43]; unknown origin, perhaps linked to Etrurian [62] | A clean (*mundau*) or cleared away valley (*accu*) [40]. Reference is probably made to a valley characterized by low stoniness, as windswept |
| 2 | Barisardo | Pizzu 'e monti | *“pìzzu”* ≡ *“pìttu”* ≡ *“pìttsu”*; from an onomatopoeic series <*p…zz>* associated to the noun <punta> [It.] = point, as well as to the verb <pizzicare> [It.] = to pinch [62] --- *“mònti”* ≡ *“mònte”*; cfr. <mons> [LAT.] = mountain, mount [42], linked to the Celtic root <MON> [62] | The mountain (*monti*) top (*pizzu*) [40], *i.e.* a sharp, steep summit morphological position |
| 3 | Arzana | Bruncu masedu | *“brùncu”* ≡ *“brùnku”* ≡ *“frùnku”* ≡ *“rùnku”;* cfr. <ρόγχος> {rònkos} [Byz.], <ῥύγχος> {rùnkos} [Gr.], <grùgno> [It.] = pig/boar snout [43]; also linked to the onomatopoeic series <*gr…gr>* [62] --- *“masédu”* ≡ *“masétu”*; cfr. <ma(n)suètus> [LAT.], <masèto> [Arag.] = tame, referred to animals and, figuratively, docile, mild [43] | A gentle (*masedu*)*,* squat (*bruncu*) mountaintop [43], *i.e.* a blunt summit morphological position, resembling the snout of a boar; a mountaintop that is smooth, easy to climb and to run across |
| 4 | Arzana | Canali enna | *“canàli”* ≡ *“kanàle”* ≡ *“kanàli”*; cfr. <canàlis> [LAT.] = canal, but also hollow, furrow, gorge [43]; from <κάννα> {kànna} [Gr.] = reed, and figuratively pipe [62], linked to <kànnu> [Asr. Bab.] [63] --- *“ènna”* ≡ *“yènna”* ≡ *“yànna”* ≡ *“gènna”;* cfr. <iànua> [LAT.] = door, access [43] | Gorge (*canali*) mouth (*enna*) |
| 5 | Baunei | Bacu sa ena | See also (accu) toponym no 1 --- *“èna”* ≡ *“vèna”* ≡ *“bèna”* cfr. <vèna> [LAT.] = vein, spring of water, in particular marsh [43] | A valley (*bacu*) characterized by a temporary pond (*ena*) [42] |
| 6 | Arzana | Coa 'e serra | *“còa”* ≡ *“kòda”*; cfr. <càuda, còda> [LAT.] = tail, queue --- *“sèrra”*; = literally, saw; by extension, a ridge jagged like a saw; cfr. <sèrra> [LAT.] = saw | The final part (*coa*) of a ridge (*serra*) |
| 7 | Arzana | Costa 'e monte | *“còsta”* ≡ *“kòsta”*; cfr. <còsta> [LAT.] = rib, but also hillside; linked to <kòsti> [anc. Slv.] = bone [43], [62] --- See also (monti) toponym no 2 | Mountain (*monte*) slope (*costa*) [40], *i.e.* reference is made to the back slope morphological position; knoll, hillock |
| 8 | Jerzu | Costa de selui | See also (costa) toponym no 7 --- *“selui”*; we guess a possible link with <sälvi> {salui, selui} [Antl.] = tree, cypress in particular [64]; cfr. <silva, sylva> [LAT.] = forest, shrub | Woody (*selui*) hillside (*costa*) |
| 9 | Arzana | Forada manna | *“forada”*; cfr. <foràtum> [LAT] = gorge, deep [43]; also linked to <faùrhs> [Got.] = furrow [62] --- *“mànna, mànnu”*; cfr. <màgnum> [LAT.] = great, large [43]; linked to <μέγας> {mégas} [Gr.], from the root <MEGH, MEG(H)E> [I.E.] [62-63] | A valley with a huge extension [10]; a large gorge |
| 10 | Barisardo | Teccu | *“tèccu”* ≡ *“tàkku”*; small dolomitic jagged plateaus; the root is probably pre-Roman [43] | Calcareous-dolomitic plateaus commonly referred by local populations as *Tacchi*, meaning heels [10] |
| 11 | Arzana | S'orgosa de su accu | *“orgòsa”*; a marshy, moist site; a pre-Roman noun [43] --- See also (accu) toponym no 1 | The marsh (*orgosa*) in the valley (*baccu*) |
| **Soil/geology category** | | | | |
| 12 | Elini | Perdarubia | *“pèrda”* ≡ *“pèdra”* ≡ *“pètra”*; cfr. <pètra> [LAT.] = stone [43]; linked to <πέτρα> {pètra} [Gr.] = stone --- *“rùbia, rùbiu”* ≡ *“rùyu”* ≡ *“rùggiu”* = red [43]; cfr. <russus> and <ruber> [LAT.], with the same meaning; linked to the roots <REUDH/RUDH> [I.E.] [62] | The red (*rubia*) stone (*perda*) [40] |
| 13 | Gairo | Baccu e praida | See also (accu) toponym no 1 --- *“pràida”* = stony (cfr. *“pèrda”* in toponym no 13) | The stony (*praida*) valley (*baccu*) [40] |
| 14 | Ilbono | Cuccuru rubiu | *“cùccuru”* ≡ *“kùkkuru”*; from the root <COCCA, CUCCA> [P.E.] = point, peak [43], [62]; the best translation of *“cùccuru”* into Italian is <cocùzzolo>, tracing back to <COCCA>, which refers to a very sharp mountain top --- See also (rubia) toponym no 12 | The red (*rubiu*), very sharp, acuminate peak (*cuccuru*) [40] |
| 15 | Lanusei | Punta perdedu | See also (perda) toponym no 12 | The stony (*perdedu*) peak (*punta,* <punta>[It.]) [40] |
| 15 | Osini | Perda morta | See also (perda) toponym no 12 | The dead (*morta,* <morta>[It.]) stone (*perda*) [42] |
| 17 | Perdasdefogu | Punta corona | *“coròna”* ≡ *“koròna”*; cfr. <κορώνη> {koròne} [Gr.] = a curved-shape object, <coròna> [LAT.] = krown, but also a cornice, a circular object | The conical-shaped (*corona*) peak (*punta,* [It.]) |
| 18 | Seui | Bruncu cogonis | See also (bruncu) toponym no 3 --- *“cogònis”* ≡ *“kogòne”* = loaf; cfr. <κόκκος> {kòkkos} [Gr.], <còccum> [LAT.] = fruit stone; by extension, a rounded, spherical object; *“kogòne”* also refers to a stale, hardened loaf [43] | The peak (*bruncu*) of smoothed pebbles (*cogonis*) [42]. In this context, the term suggests the presence of rounded, loaf-shaped stones or pebbles |
| 19 | Talana | Bacu nieddu | See also (accu) toponym no 1 --- *“nièddu”* ≡ *“nigèddu”* = black; cfr. <nigellus> [LAT.] = blackish [43], <nìger> [LAT.] = black | The black (*nieddu*) valley (*bacu*) [40] |
| 20 | Tertenia | Monte ferru | See also (monti) toponym no 2 --- *“ferru”*; cfr. <fèrrum> [LAT.] = iron [43] | The iron (*ferru*) mountain (*monte,*<monte>[It.]) [42] |
| 21 | Tortolì | Baccu 'e tonara | See also (accu) toponym no 1 --- *“tònara”* ≡ *“tònneris”*; dolomitic cones, with fantastic forms, resulting from karstic erosion of calcareous massifs, tyical of Ogliastra region; the root is probably pre-Roman [43] | The valley (*baccu*) of “tonneris” (*tonara*)*,* conical-shaped dolomitic formations |
| 22 | Ulassai | Monte perdalonga | See also (perda) toponym no 12 | The mountain (*monte, <*monte*>* [It.]) of the long (*longa*) stone (*perda*) [40] |
| **Morphology/fauna category** | | | | |
| 23 | Urzulei | Arcu correboi | *“àrcu”*≡*“àrku”*; cfr. <àrcus> [LAT.] = bow, as well as arch --- *“còrre”* ≡ *“kòrru”*; cfr. <còrnu> [LAT.] = horn; from the root <KER> [I.E.] = protuberance, protrusion [62] --- *“bòi”* ≡ *“bòe”* ≡ *“òe”*; cfr. <bòs> [LAT.] = ox; from the root <GwOUS> [I.E.] = bovine [62] | A mountain pass (*arcu*) characterized by an ox (*boi*) horn (*corre*) shape [10]; the “arc” is a peculiar morpho-geological formation that frequently occurs in calcareous rocks where karstic processes are intensive |
| 24 | Villagrande | Arcu is colorvos | *“colòrvos”* ≡ *“kolòvra”* ≡ *“kolòvru”*; cfr. <còluber, còlubra> [LAT.] = grass-, water-snake | The arc (*arcu*) of the snakes (*colorvos*) [43] |
| 25 | Jerzu | Baccu de ois | See also (accu and boi) toponym no 1 and 23 | The oxen (*ois*) valley (*baccu*) [40] |
| 26 | Lanusei | Arcu su sinsulu | *“sìnsulu”* ≡ *“sìntsulu”* ≡ *“tsìntsulu”* = jujube; cfr. <zìziphus> [LAT.], <ζίζυφον> {zìzufon} [Gr.] | The arc (*arcu*) of the jujube (*sinsulu*) |
| 27 | Osini | Bruncu de gespis | See also (bruncu) toponym no 3 --- *“géspis”* ≡ *“gèspe”* ≡ *“èspe”* ≡ *“èspu”*; cfr. <vèspa> [LAT.] = wasp; likely linked to the missed <vopsa> from German and Baltic areas [62] | The peak (*bruncu*) of the wasps (*gespis*) [40] |
| 28 | Seui | Monte 'e s'ebba | See also (monti) toponym no 2 --- *èbba”* ≡ *“ègwa”* ≡ *“ègua”*; cfr. <èquus> [LAT.] = horse; from <EKWOS> [I.E.], also linked to the Sanskrit <AÇVAS> [62] | The broodmare (*ebba*) mountain (*monte*, <monte> [It.]) [40] |
| 29 | Talana | Bruncu abes | See also (bruncu) toponym no 3 --- *“àbe”* ≡ *“àpi”*; cfr. <àpis> [LAT.] = bee | The peak (*bruncu*) of the bees (*abes*) [40] |
| 30 | Tertenia | Taccurrulu | See also (teccu) toponym no 10 | The plateau of the owl (*taccurrulu,* ***E.n.A.***) [42] |
| 31 | Tortolì | Monte attu | See also (monti) toponym no 2 --- *“àttu”* ≡ *“gàttu”*; cfr. <càttus> [LAT.] = cat | The cat (*attu*) mountain (*monte*, <monte> [It.]) [42] |
| **Morphology/vegetation category** | | | | |
| 32 | Triei | Azza selimba | *“àzza”*≡*“àtta”*≡*“àttsa”*; cfr. <àcies> [LAT.] = cutting edge; by extension, a craggy, sharp mountain flank | The craggy mountain shoulder (*azza*) characterized by the presence of carob (*silimba*) [40]. The carob is the fruit of the carob tree (Ceratonia siliqua L.) |
| 33 | Ulassai | Serra su suergiu | See also (serra) toponym no 6 --- *“suèrgiu”* ≡ *“subèriu”* ≡ *“suèrgu”* = cork; cfr. <sùber> [LAT.] = ork, as well as cork oak | The jagged peak (*serra*) of the cork (*suergiu*) [40]. The cork is the bark of the cork oak (Quercus suber L.) |
| 34 | Urzulei | Bacu gadalattu | See also (accu) toponym no 1 | The valley (*bacu*) of the helichryse (*gadalattu*, ***E.n.A.***) [42]. **H*elichrysum Italicum* Roth** (Don) |
| 35 | Villagrande | Bruncu pauli gosti | See also (bruncu) toponym no 3 --- *“paùli”* ≡ *“padùle”* ≡ *“paùle”* = marsh; cfr. <pàlus> [LAT.] = marshland; linked to <πλάδος> {plàdos} [Gr.] = extreme moistness, aquosity [62], referable to the root <PELD> [I.E.] [63] | A squat mountain summit (*bruncu*) characterized by the presence of temporary pond or marshes (*pauli*) and Montpellier maples (Acer Monspessulanum L.) or box trees (Buxus sempervirens L.) (*gosti*, ***E.n.A.***) [42] |
| 36 | Baunei | Funtana bacu arangius | *“funtàna”*≡*“puntàna”* = spring, fountain; cfr. <fòns> [LAT.] = spring, as well as spring water --- See also (accu) toponym no 1 --- *“aràngiu”* ≡ *“aràntsu”* = orange; cfr. <nàrang> [Pers.] | A spring (*funtana*) in the valley (*bacu*) of the orange trees (*arangius*) (Citrus sinensis (L.) Osbeck.) [42] |
| 37 | Girasole | Canale murdegus | See also (canali) toponym no 4 --- *“murdègus”* ≡ *“mutèclu”* ≡ *“mutrècu”* = various cistus species; a pre-Roman noun, related to the Etrurian <mùtuka> [43]; | A narrow-stretched valley (*canale*) characterized by the presence of Montpellier rock rose (*murdegus*) (*Cistus monspeliensis* L.) [40] |
| 38 | Jerzu | Bruncu de issarbussus | See also (bruncu) toponym no 3 | The peak (*bruncu*) of the asphodels (*issarbussus,* ***E.n.A.***) (*Asphodelus microcarpus* Salzm. et Viv.) [40] |
| 39 | Lanusei | Genna ortiga | See also (enna) toponym no 4 | A mouth (*genna*) with nettle (*ortiga,* assonant to <ortica>[It.]) (Urtica dioica L.) [40] |
| 40 | Loceri | Monte lua | See also (monti) toponym no 2 --- *“lùa”* = euphorbia; cfr. <lùes> [LAT.] = venomous liquid, contagion; Wagner [43] argues that *“lùa”* is utilized to poison water, then to knock out fishes | The mountain (*monte*) of the tree spurge (*lua*) (*Euphorbia dendroides* L.) [40] |
| 41 | Osini | Cuccuru ollustincu | See also (cuccuru) toponym no 14 --- *“ollustìncu”* ≡ *“ollustìnci”* ≡ *“òdzu ‘e listìnku”* = oil from lentisk, once utilized for lamps; cfr. <òleus> [LAT.] = oil, and <lentìscus> [LAT.] = lentisk [43] | The peak (*cuccuru*) of the oil lentisk (*Pistacia lentiscus* L.) [40] |
| **Vegetation category** | | | | |
| 42 | Osini | Figu niedda | See also (nieddu) toponym no 19 | Black (*niedda*) fig (*figu,* assonant to <fico>[It.]) (*Ficus carica* L.) [40] |
| 43 | Perdasdefogu | Is lionagis | *“lionàgis”* ≡ *“lisàndru”* ≡ *“olisàndru”* = oleander; cfr. <(h)olus atrum> [LAT.] = *Smyrnium olusatrum* L. | The oleanders (*lionagis*) (*Nerium oleander* L., or *Smyrnium olusatrum* L.) [42] |
| 44 | Talana | Ponte tipparo | *“tìpparo”* ≡ *“tsìppiri”* = rosemary; likely a Punic language relict [43] | Rosemary (*tipparo*) (Rosmarinus officinalis L.) bridge (*ponte,* <ponte>[It.]) [42] |
| 45 | Tertenia | Cuile lionis | *“cuìle”* ≡ *“kubìle”*; literally, den, burrow; but also stockyard, sheep fold; by extension, bed, field; cfr. <cubìle> [LAT.] = couch, pallet, bed --- *“liònis”* ≡ *“olidòne”* ≡*“lidòne”* = arbutus, strawberry tree; cfr. <unèdo(nis)> [LAT.] = *Arbutus unedo* L. | The fold (*cuile*) of the strawberry trees (*lionis*) (Arbutus unedo L.) [40] |
| 46 | Tortolì | Cugumeru |  | Cucumber (*cugumeru*, assonant to <cocomero> [It.]) (Cucumis sativus L.) [40] |
| 47 | Triei | Riu arridellu |  | The river (*riu*, assonant to <rio> [It.]) of the narrow leaf phillyrea (*arridellu*, ***E.n.A.***) (*Phillyrea angustifolia* L.) [43] |
| 48 | Ulassai | Su pirastu |  | The European wild pear (p*irastru*, assonant to <perastro> [It.]) (Pyrus pyraster (L.) Burgsd) [40] |
| 49 | Ussassai | Orgiu fresu | *“òrgiu”* ≡ *“òrju”* = barley; cfr. <hòrdeum> [LAT.] = barley --- *“frèsu”*; from *“fresare”* = to chap, to crack; cfr. <frèndere> [LAT.] = to grind | The cut (*fresu*) barley (*orgiu*) (Hordeum vulgare L.) [43]; more probably, milled (*fresu*) barley, barley flour |
| 50 | Urzulei | Campu 'e sa murta | *“mùrta”* = myrtle; cfr. <μύρτος> {mùrtos} [Gr.], <mùrta, mùrtos> [LAT.] | The field (*campu*, assonant to <campo> [It.]) of myrtles (*murta*) (Myrtus communis L.) [40] |
| 51 | Villagrande | Riu sos sammucheddos | *“sammucchèddos”* ≡ *“sabùkku”* ≡ *“samùkku”* = elder; cfr. <sambùcus, sabùcus> [LAT.] | The river (*riu*, assonant to <rio> [It.]) of the small elders (*sammuccheddos*) (*Sambucus nigra*L.) [42] |
| 52 | Barisardo | Teria |  | Thorny broom (*teria*, ***E.n.A.***) (*Calicotome spinosa* (L.) Link) [42] |
| **Soil cover/land uses category** | | | | |
| 53 | Lanusei | Funtana 'e padenti | *“padènti”* ≡ *“patènte”* = “public or private oak wood where pigs are allowed to eat acorns” [43]; cfr. <pàtens> [LAT.] = open, free access | The spring (*funtana*) in the oak wood where swine are allowed to eat acorns (*padenti*) [40] |
| 54 | Lotzorai | Bingiamanna | *“bìngia”* ≡ *“bìndza”* ≡ *“vindza”*; cfr. <vìnea> [LAT.] = vineyard; from the Mediterranean root <(w)ÒINOS>, hence <οἶνος> {òinos} [Gr.] [62] --- See also (manna) toponym no 9 | The great (*-manna*) vineyard (*bingia-*) [40] |
| 55 | Seui | Riu girinedda |  | The river (*riu*, assonant to <rio> [It.]) in the last part of an uncultivated field where the plough turns back (*girinedda,* ***E.n.A.***) [40] |
| 56 | Talana | Bacu orgiares | See also (accu) toponym no 1 --- *“orgiàres”* ≡ *“ordzàle”* = soil suitable for barley cropping; cfr. *“òrgiu”* | A valley (*bacu*) characterized by soils suitable for the barley production (*orgiares*) [42] |
| 57 | Tortolì | Casa ortalis mannu | *“ortàlis*, derivative of *“òrtu” ---* See also (manna) toponym no 9 | The house (*casa,* <casa> [It.]) in the great (*mannu*) garden (*ortalis*) [40] |
| 58 | Ulassai | Cuccuru is argiolas | See also (cuccuru) toponym no 14 --- *“argiolas”* ≡ *“arjòla”* ≡ *“ardzòla”* = threshing area; cfr. <arèola> [LAT.] = small open space, court | The sharp peak (*cuccuru*) of the threshing area (*argiolas*) [40] |
| 59 | Urzulei | Bruncuolevani | See also (bruncu) toponym no 3 | The squat peak (*bruncu*) of the bovine pasture (*olevani,* ***E.n.A.***) [10] |
| 60 | Villagrande | Ortu eliai | *“òrtu”*; cfr. <hortus> [LAT.] = enclosure, garden, park; also cfr. <χόρτος> {chòrtos}[Gr.], from the root <GHÒRTO> once spread in Oscan-Umbrian and Celtic areas [62] --- *“èliai”* ≡ *“èlike”* ≡ *“èlige”* = holm oak; cfr. <ìlex> [LAT.] | The garden (*ortu*) of the Holm oak (*eliai*) (*Quercus ilex L.*) [42] |

^a^“de, ‘e” = of; “ s’, su, sa, is, sos” = the.

^b^*“…”* denote Sardinian words (SL); <…> denote words in other languages, as well as root logatomes and onomatopoeic phonemes; ≡ denotes a linguistic equivalent variant; cfr.: confer (compare); {} denotes a transliteration; [Gr.]: Greek; [Byz.]: Byzantine; [LAT.]: Latin; [It.]: Italian; [Arag.]: Aragonese; [Asr. Bab.]: Assyro-Babilonian; [anc. Slv.]: ancient Slavic; [Antl.]: Anatolian; [Got.]: Gothic; [I.E]: Indo-European; [P.E.]: Paleo-European; [Pers.] Persian; = denotes the current meaning; --- separates the phonetic transcription of two terms belonging to the same toponym.

^c^<…> [It.]: Italian noun; ***E.n.A.***= Etymon not Attested by current bibliography.

**References**

1. Calonghi F. Dizionario Latino-Italiano. Torino: Rosenberg & Seller ed; 1962.
2. Devoto G. Avviamento alla etimologia Italiana. Dizionario etimologico. Firenze: Le Monnier ed; 1968.
3. Rocci L. Vocabolario Greco-Italiano. Roma: Società Editrice Dante Alighieri Srl ed; 2011.
4. Rocchi L. Il dizionario Turco-Ottomano di Arcangelo Carradori (1650). Trieste: EUT-Edizioni, Università di Trieste ed; 2011.
